# Biochemical Insights into the Ability of *Lemna minor* L. Extract to Counteract Copper Toxicity in Maize

**DOI:** 10.3390/plants11192613

**Published:** 2022-10-06

**Authors:** Begoña Miras-Moreno, Biancamaria Senizza, Luca Regni, Ciro Tolisano, Primo Proietti, Marco Trevisan, Luigi Lucini, Youssef Rouphael, Daniele Del Buono

**Affiliations:** 1 Department for Sustainable Food Process, Università Cattolica del Sacro Cuore, Via Emilia Parmense 84, 29122 Piacenza, Italy; 2 Dipartimento di Scienze Agrarie, Alimentari e Ambientali, Università degli Studi di Perugia, Borgo XX Giugno 74, 06121 Perugia, Italy; 3 Department of Agricultural Sciences, University of Naples Federico II, 80055 Portici, Italy

**Keywords:** Duckweed, copper toxicity, photosynthesis, plant growth, SOD, APX, CAT, oxidative status, bioactive metabolites

## Abstract

Metal trace elements (MTE) can damage crops if present in excessive amounts in the environment. This research investigated the effect of a plant extract of an aquatic species, *Lemna minor* L. (duckweed) (LE), on the ability of maize to cope with copper (Cu) toxicity. LE reversed the effects of Cu^2+^ on photosynthetic activity (Pn), evapotranspiration (E), stomatal conductance (gs), sub-stomatal CO_2_ concentration (Ci) and biomass which did not differ from the untreated controls. LE did not regulate the amount of copper in maize leaves, but compared to Cu-treated samples, the extract decreased the hydrogen peroxide (H_2_O_2_; −26% on average) and malondialdehyde (MDA; −47% on average) content, regardless of the dosage applied. Furthermore, the activity of antioxidant enzymes superoxide dismutase (SOD), ascorbate peroxidase (APX) and catalase (CAT) was significantly increased by LE compared to samples treated with Cu alone. Untargeted metabolomic profiling revealed that LE activated maize secondary metabolism, eliciting the content of non-enzymatic antioxidants (flavonoids, glutathione and glutathione-related compounds, tocopherols and tocotrienols) and modulating plant stress-related hormones (brassinosteroids and ABA derivatives). The results of this study are promising and pave the way for using duckweed as a biostimulant to trigger beneficial effects in maize and increase its resistance to MTEs.

## 1. Introduction

Many environmental problems are associated with the dispersion and subsequent accumulation of excessive amounts of metal trace elements (MTE) in the environment. MTE include toxic metalloids and heavy metals, and copper (Cu) is in this category [1]. In addition, Cu is a concern due to its continuous release into the environment as a result of anthropic activities [2]. For instance, the contribution of agriculture to the dispersion of Cu into the environment is significant as cropping systems use large quantities of fertilizers, fungicides, and sewage sludge, all of which contain considerable quantitative of this element [2]. As a result, Cu accumulates in the soil of cultivated areas with the risk of reaching crops [3]. 

Nonetheless, plants need trace amounts of Cu in cationic form because it is essential for their metabolism, as it participates in crucial processes that enable plants to achieve normal growth and development [4]. For example, this element is required by cells for electron transport in respiration and photosynthesis and plays a signalling role in protein transcription and Fe mobilisation [5,6]. The Cu concentration in leaf tissues varies from 5 to 20 µg g-1 dry weight, depending on the species [7].

In contaminated environments, Cu can cause adverse effects on plants, such as nutritional disorders and physiological, morphological and biochemical alterations [3]. In addition, Cu can inhibit plant growth already at very low concentrations [3]. Cu can affect photosystem II (PSII), decrease the content of photosynthetic pigments, and degrade thylakoid membranes [8,9]. In addition, Cu can hinder the ability of plants to assimilate CO_2_, which is associated with a decrease in stomatal conductance [7]. Due to these effects, plants may exhibit stunted growth, biomass decline and death [9]. 

Cu can also induce oxidative stress through the Haber-Weiss and Fenton reactions, leading to the overproduction of reactive oxygen species (ROS) [4], which can be very harmful to plants due to their ability to degrade membranes, lipids, proteins, and nucleic acids [2,10]. However, plants possess enzymes and antioxidant molecules that can contain the deleterious effects of ROS, if the cellular capacity to cope with oxidative damage is not exceeded. [4]. 

Given the concern about the effects of abiotic stresses on crop health and productivity, particular attention is being paid to sustainable technologies that can increase plant resistance. In this regard, biostimulants attract particular interest because they can increase crop productivity and enhance their ability to resist abiotic stresses [11,12]. Biostimulants are a heterogeneous class of natural products that can also improve crop nutrition and the quality of the end products [12]. Furthermore, biostimulants are distinguished from fertilisers and plant protection products. [13]. Biostimulants can be obtained from various raw materials belonging to different families, such as humic and fulvic substances, protein hydrolysates and amino acids, seaweed and plant extracts, chitin and chitosan derivatives and beneficial microorganisms [14].

Regarding the use of biostimulants to improve crop tolerance to abiotic stress, their effectiveness has been demonstrated in plants subjected to drought, salinity and temperature extremes [14,15,16]. Furthermore, biostimulants have been reported to help plants to grow in polluted environments, such as those containing MTE, by improving plant tolerance [17]. For instance, biostimulated maize plants minimized the effects of cadmium toxicity by inducing genes related to antioxidant defences, maintaining biomass production, hormone homeostasis and photosynthesis [17]. Moreover, seed-priming with biostimulants increased maize resistance to abiotic stress by influencing the hormone signalling pathways and stimulating antioxidant defences [18].

Despite the numerous products available on the market, the search for new plant extracts with a biostimulant action is steadily increasing due to their biocompatibility [19]. In this context, *Lemna minor* L. (called duckweed), a free-floating wild aquatic species, is receiving some interest. Duckweed is widespread in several continents, characterised by a rapid growth rate and adaptable to even unfavourable environmental conditions (wide temperature and pH range); moreover, it is easily cultivable under controlled conditions [20,21]. Furthermore, this plant shows antioxidant properties that have made it the subject of numerous studies [20]. It has recently been shown that an extract of duckweed exerted a biostimulating action on maize and olive grown under optimal conditions [20,21]. In particular, the extract exhibited a high content of phytochemicals with biostimulant effects, such as phenolics and glucosinolates, responsible for promoting maize germination, biomass production and vigor index [20], as well as photosynthetic performance, and pigment content in olive trees [21].

In light of these premises, the objective of this study was to verify the possible beneficial effects of a duckweed extract on maize seedlings treated with 20 µM Cu. To this end, some extract concentrations were tested, and the physiological and biochemical parameters, oxidative status and antioxidant defences of the treated crop were investigated. Furthermore, an untargeted metabolomic approach was also carried out to shed light on the mechanisms underlying the attenuation of Cu-related stress in maize in a complementary manner.

## 2. Results

### 2.1. Leaves Gas Exchanges and Plant Biomass

**Figure 1:**
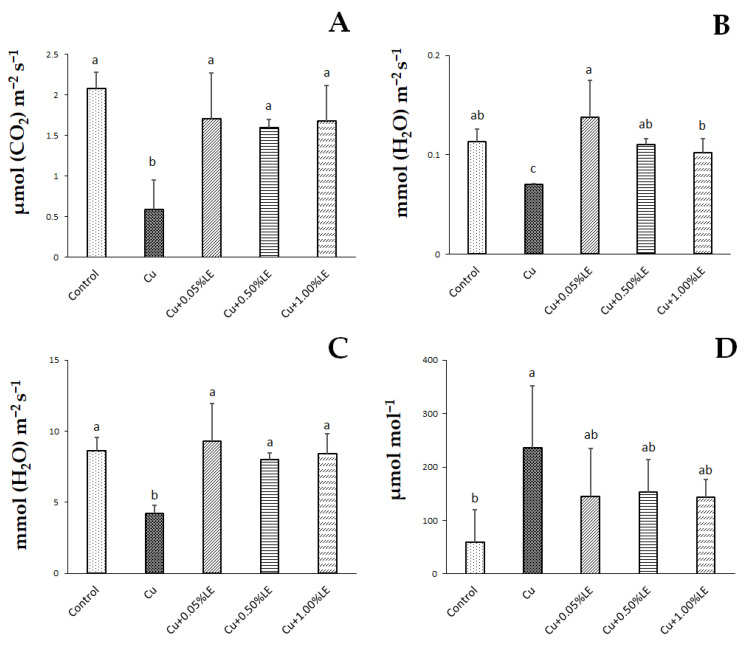
Means value of leaf net photosynthesis (**A**) (μmol (CO_2_) m^−2^ s^−1^), leaf transpiration rate (**B**) (mmol (H_2_O) m^−2^ s^−1^), stomatal conductance (**C**) (mmol (H_2_O) m^−2^ s^−1^) and sub-stomatal CO_2_ concentration (**D**) (μmol mol^−1^) recorded in control maize, samples treated with Cu alone and in combination with 0.05%, 0.50% and 1.00% LE. Bars indicate standard deviations (SD). Values with different letters were significantly different (*p* < 0.05), as indicated by the one-way ANOVA followed by Duncan’s new multiple range test.

In maize plants treated with Cu alone, a decrease in net foliar photosynthesis (Pn) was observed compared to control samples (Figure 1). The decrease in Pn in Cu-stressed plants was accompanied by an increase in sub-stomatal CO_2_ concentration (Ci) and a decrease in stomatal conductance (gs) (Figure 1). The latter effect was also accompanied by a significant reduction in leaf transpiration rate (E) (Figure 1). On the contrary, the duckweed extract stimulated at all the concentrations investigated in Cu-treated samples, Pn and restored gs, E and Ci to values that did not differ statistically from those shown by the control samples (Figure 1).

Samples treated with Cu alone showed reductions in plant growth in terms of shoot and root length and shoot and root fresh weight (Table 1). However, when maize was treated with the duckweed extract, the Cu-induced reduction in growth was firmly contained or even eliminated. In fact, the samples treated with LE, regardless of the dosage applied, showed shoot and root lengths significantly higher than those of samples treated with Cu alone, which did not significantly differ from that of the control samples. Finally, a similar trend was also found for the shoot and root fresh weight, except for the treatment with Cu + 0.50% LE that stimulated these two parameters to values even higher than those shown by the control samples. 

### 2.2. Copper (Cu), Hydrogen Peroxide (H_2_O_2_) and Malondialdehyde (MDA) Contents in Maize Leaves

The Cu content was assessed in maize samples subjected to different treatments (Table 2). In general, all Cu-treated samples showed approximately twice the copper content of the control samples (Table 2). In addition, the duckweed extract did not affect the amount of copper present in all Cu-treated samples.

Concerning the H_2_O_2_ content, the samples treated with copper without adding LE showed the highest value of hydrogen peroxide (Table 2). In contrast, some differences were found when maize seeds were treated with LE. In particular, Cu + 0.50% LE showed H_2_O_2_ values that did not differ statistically from the control samples. Furthermore, the other LE treatments showed significantly lower H_2_O_2_ values than the maize samples treated with Cu alone, although they were significantly higher than those of the control samples (Table 2). 

Regarding MDA, the samples treated with Cu alone showed a significantly higher value of this lipid peroxidation product than the control samples. In addition, the MDA content found in the samples treated with copper alone was significantly higher than that in those treated with LE, regardless of the dosage applied (Table 2). Moreover, Cu + 0.05% LE and Cu + 0.50% LE showed MDA values that did not differ statistically from those of the control samples. Finally, Cu + 1.00% LE treatment showed a higher MDA content than all other LE-treated samples, but it was statistically lower than the value shown by the samples treated with Cu alone (Table 2). 

### 2.3. Activity of the Antioxidant Enzymes SOD, APX and CAT

**Figure 2:**
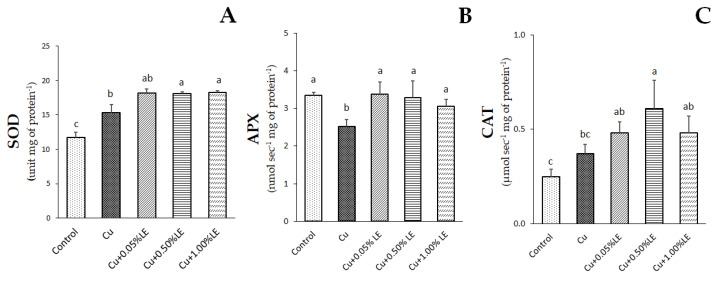
Superoxide dismutase (SOD—**A**), ascorbate peroxidase (APX—**B**) and catalase (CAT—**C**) activity recorded in control maize, samples treated with Cu alone and in combination with 0.05%, 0.50% and 1.00% LE. Bars indicate standard deviations (SD). Values with different letters were significantly different (*p* < 0.05), as indicated by the one-way ANOVA followed by Duncan’s new multiple range test.

The activity of three antioxidant enzymes, SOD, CAT and APX, which protect plants from oxidative stress, was studied (Figure 2). As for the superoxide dismutase (SOD), the enzyme activities recorded in the samples treated with Cu + 0.50% LE and Cu + 1.00% LE were the highest and significantly different from those of the control samples (Figure 2). As for Cu + 0.05% LE, the enzyme activity was not statistically different from that of the samples treated with Cu alone, although it was higher than that of the control samples. 

Concerning ascorbate peroxidase (APX), Cu significantly reduced the activity of this enzyme when administered alone. However, when maize seeds were treated with LE, regardless of the dosage applied, APX activity returned to values that did not differ significantly from those shown by the control samples.

In the case of catalases (CAT), the trend differed from that recorded for the two previous enzymes. In particular, all treatments, except those with Cu alone, resulted in a significant induction of CAT activity compared to the control samples. However, only the treatment with Cu + 0.50% LE was statistically different from the results recorded for the samples treated with Cu alone.

### 2.4. Untargeted Metabolomic Profile of Maize Samples

An untargeted metabolomic approach was applied to study the maize response to LE application at a molecular level under Cu stress conditions. Using the comprehensive database PlanCyc 12.6, more than 1500 compounds were putatively annotated (Supplementary Table S1) and used for the following multivariate statistical analysis.

**Figure 3:**
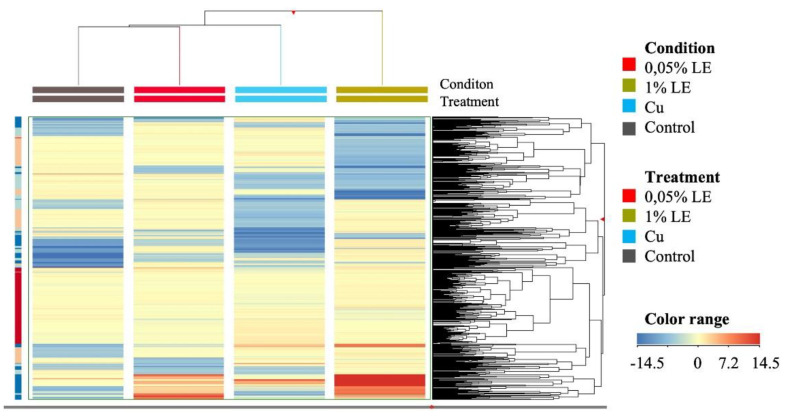
Unsupervised hierarchical cluster analysis carried out from UHPLC-ESI/QTOF-MS metabolomic analysis of maize plants Cu-stressed and treated with Cu + 0.05% LE and Cu + 1.00% LE. The fold-change based heat map was used to build hierarchical clusters (linkage rule: Ward; distance: Euclidean).

Firstly, unsupervised hierarchical cluster analysis was used to understand better the similarities and differences in maize metabolomic signatures among the treatments. As shown by the heat map (Figure 3), two main clusters were generated from the metabolomic profile of the different samples. The first cluster was represented by the highest LE concentration (Cu+1.00 % LE), while the second cluster included Cu + 0.05% LE, Cu-stressed and control plants (Figure 3). 

**Figure 4:**
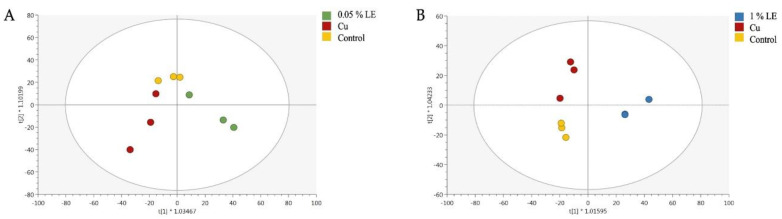
Orthogonal projection to latent structures discriminant analysis (OPLS-DA) supervised of maize plants treated with 0.05% LE (**A**) and 1.00% LE (**B**) treatments. The metabolomic dataset produced through UHPLC-ESI/QTOF-MS was Pareto scaled and then used for the multivariate OPLS-DA modeling (R^2^Y = 0.71 Q^2^Y = 0.41 (A) and R^2^Y = 0.88 Q^2^Y = 0.47 (B)).

Afterwards, these results were further confirmed by the supervised OPLS discriminant analysis. The OPLS-DA score plot, built considering the Cu + 0.05% LE and Cu + 1.00% LE (Figure 4), separated treated plants from untreated and Cu-stressed plants, as suggested by the HCA (Figure 3). 

The VIP approach was used to identify the discriminant compounds modulated by the treatments compared to the control. Over 100 compounds were found as the most discriminant markers (VIP score >1.2). A detailed list of discriminant compounds and their VIP scores for the 0.05% LE and 1.00% LE treatments can be found in Supplementary Tables S2 and S3, respectively. 

**Figure 5:**
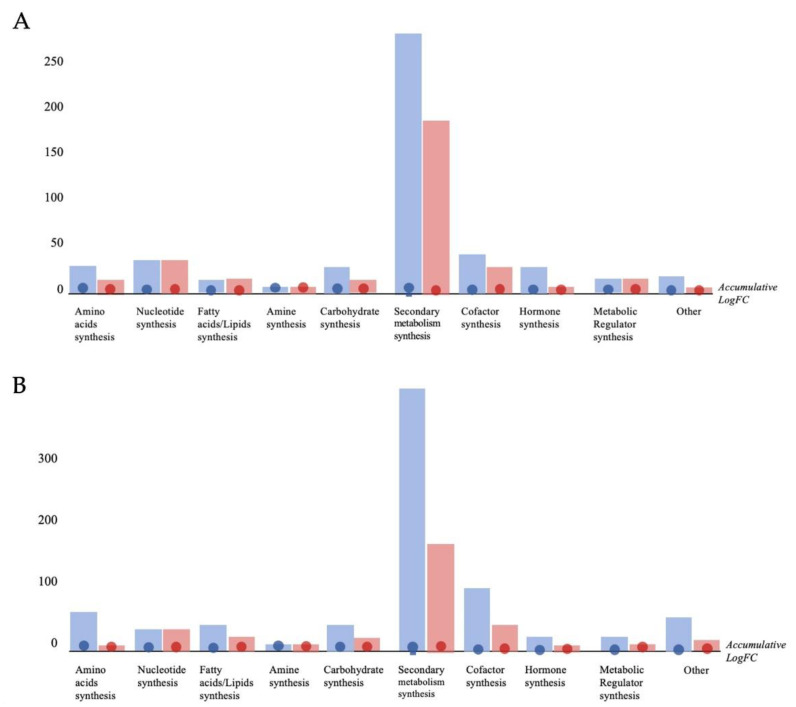
Metabolic processes modulated in maize plants treated with 0.05% LE (**A**) and 1.00% LE (**B**) treatments. VIPs markers were loaded into the PlantCyc Pathway Tool (https://www.plantcyc.org/ Plant Metabolic Network; accessed on 20 November 2021). The x axis represents each set of metabolic subcategories, while the y axis corresponds to the accumulative log fold change (FC). The large dots represent the average (mean) of all FCs for the different metabolites in the class, while the small dots represent the individual log FC.

Figure 5 summarizes the discriminant compounds (VIP markers) classified in the biochemical classes and depicts the general trend in the modulation of biosynthetic processes by Cu-stressed, Cu + 0.05% LE and Cu + 1.00% LE based on changes in metabolites accumulation compared to the control. 

Cu-stress triggered a plant response mainly involving secondary metabolism. This effect on plant defense appeared to be enhanced by the duckweed extract, which stimulated the accumulation not only of secondary metabolites but also of antioxidant compounds (Figure 5; Supplementary Tables S2 and S3). Moreover, the effect seemed to be more significant for 1.00% LE than for 0.05% LE, in agreement with multivariate statistics.

The combination of Cu + LE strongly elicited phenylpropanoids (and flavonoids in particular), which are powerful antioxidants and direct ROS scavengers [22], to a greater extent than Cu stress alone. In addition, nitrogen-containing secondary metabolites (glucosinolates) and terpenes were positively regulated. 

In addition, the application of LE extracts was not limited to the elicitation of secondary metabolism but also provoked a positive modulation of several phytohormones related to stress tolerance (i.e. ABA, brassinosteroids and salicylate-related compounds). 

Oxidative stress seemed to be activated, as several compounds such as GSSG, caffeoylserotonin or arginino-succinate were strongly accumulated in the case of 1.00% LE and, to a lesser extent, in the case of 0.05% LE.

## 3. Discussion

Farming systems are increasingly confronted with various abiotic stresses which can reduce crop productivity. Among the abiotic stressors, drought, salinity, and heavy metals impact crop species [3]. For instance, many studies have documented that Cu at toxic levels can inhibit plant growth, induce oxidative damage and negatively affect photosynthetic activity, chlorophyll biosynthesis, and plant mineral nutrition [3]. 

Our experiments showed reductions in photosynthetic activity in plants treated with Cu alone, and the results obtained agree with other studies that have attributed this effect to structural impairments of the photosynthetic apparatus [3]. In plants subjected to abiotic stress, the reduction in photosynthetic activity may be accompanied by an increase in sub-stomatal CO_2_ concentration that causes stomatal to close, thus decreasing stomatal conductance and evapotranspiration [23,24]. The ability of copper to influence the sub-stomatal CO_2_ concentration suggests that the reduction in photosynthetic activity was mainly caused by non-stomatal effects and could result from specific or more general damages to photosystems [25]. In particular, it has been reported that an excess of Cu could damage both photosystems, but PS II is the most sensitive to copper exposure [26]. A decrease in photosynthetic activity in Cu-treated plants was also observed in cucumber [27] and pea plants [28].

Furthermore, toxic concentrations of Cu in barley plants can reduce photosynthetic activity, probably due to mesophyll limitations and chloroplast membrane damage [29]. In this context, biostimulants are considered a useful agronomic tool to increase crop productivity and mitigate the deleterious effects of abiotic stresses [30]. Our experiments revealed that the increases in photosynthetic activity found in maize treated with the duckweed extract and Cu were associated with increases in the stomatal conductance, suggesting that the extract also improved photosynthesis by positively influencing the stomatal aperture. In line with these results, photosynthetic activity and stomatal conductance increased in broccoli and olive after treatment with biostimulants [31,32]. Moreover, an increase in photosynthetic activity was observed in hibiscus and *Euphorbia × lomi* plants in response to the treatment with a biowaste [33], in greenhouse jute treated with a commercial biostimulant derived from a tropical plant (PE; Auxym ®, Italpollina, Rivoli Veronese, Italy) [34] and in lettuce and strawberries treated with borage extracts and Actiwave® [35,36]. 

The beneficial effect of the duckweed extract on the photosynthetic activity of Cu-treated plants can also explain the positive effects on plant growth and biomass production compared to samples treated with copper alone. Recently, an extract of Fucus ssp, *Ascophyllum nodosum* and *Pelvetia canaliculata* was shown to positively influence barley grown in Cu-contaminated soil, counteracting most of the negative effects of copper on plant growth [37]. Moreover, silymarin enriched with a maize grain extract attenuated the effects of cadmium stress in *Z. mays*, promoting plant growth and biomass accumulation [17]. 

In general, it has been hypothesized that the capacity of biostimulants to counteract the adverse effects exerted by copper, or more in general by heavy metals, resides in the ability of these materials to reduce the amount of copper taken up by plants or to activate tolerance mechanisms linked to the induction of antioxidant enzymes or molecules [37]. To shed light on this point, we first analysed the copper content in maize shoots biostimulated or not with the duckweed extract; then, we monitored the oxidative status of the plant by measuring the hydrogen peroxide (H_2_O_2_) and MDA content and the activity of some antioxidant defensive enzymes.

The extract did not influence the copper content in shoots; in fact, the copper-treated samples, both biostimulated and non-biostimulated, showed approximately the same amount of Cu, which was generally twice that found in the control samples. Concerning H_2_O_2_, a significant increase in this ROS was found in samples treated with copper alone, but LE strongly reduced its content. Since H_2_O_2_ can impair cell function when it accumulates under stress [38], the effect induced by the duckweed extract was the stimulation of protective mechanisms to contain this oxidant. 

In general, abiotic stresses can damage membranes due to the overproduction and accumulation of ROS [39], which are reactive toward a plethora of molecules, and MDA, a lipid degradation product, can accumulate when the cell undergoes oxidative stress [39]. Our experiments showed that the duckweed extract reduced the MDA concentration in maize subjected to the different treatments. These results agree with those obtained for the hydrogen peroxide content and highlight the ability of LE to improve the oxidative status of maize plants. Other studies have demonstrated the protective action of biostimulants against heavy metals or in mitigating their phytotoxic effects [37,40]. 

Biostimulants to protect crops from the deleterious effects of heavy metals can induce the activity of enzymes involved in oxidative stress responses. To this end, we studied the activity of SOD, APX and CAT in samples subjected to the various treatments. SOD acts by disproportionating O_2_^−^ to O_2_ and H_2_O_2_, which in turn is mainly removed by APX and CAT [30]. SOD is the first defence against oxidative perturbations [39]; APX is the main enzyme that removes H_2_O_2_ in the chloroplast and uses ascorbic acid as the electron donor [41]; CAT is found in peroxisomes and glyoxisomes and acts in removing H_2_O_2_ in leaves [42]. Our results showed that in samples grown with copper alone, the only enzyme with higher activity than the control was SOD, while CAT showed no significant difference and APX decreased its activity. The induction of SOD in response to copper treatments has been documented in other studies and is functional in coping with stress conditions [9,43]. In contrast, the significant decrease in APX activity may justify the accumulation of MDA, as this enzyme is considered particularly important in removing H_2_O_2_ [9]. Such an impact of Cu on antioxidant defences may lead to the accumulation of hydrogen peroxide and MDA, showing that the cells were under oxidative stress [37,44]. In contrast, when maize was biostimulated with the duckweed extract, SOD and CAT activities were significantly higher than those of the control samples and the APX activity returned to values comparable to those of the untreated samples. The activation of SOD and CAT is relevant and the induction of these enzymes has been correlated with the copper tolerance of *Prunus cerasifera* and other species to heavy metals [45,46]. These results reveal that among the protective mechanisms against redox imbalance, duckweed extract activated antioxidant enzymes or prevented the decrease in their activity, thus containing H_2_O_2_ and MDA accumulation. This agrees with the literature, which suggests that biostimulants can increase crop tolerance to abiotic stresses by stimulating antioxidant responses [15,30,47].

In addition to the enzymatic antioxidant defence system, plants possess a non-enzymatic antioxidant mechanism to cope with ROS generation, which includes glutathione and flavonoids, among other metabolites [48]. In our study, the metabolic pathway analysis revealed that the application of LE activated the non-enzymatic antioxidant system in maize plants. Indeed, our results showed the involvement of several antioxidant compounds (i.e. flavonoids) and the activation of detoxification processes involving glutathione-related compounds. Moreover, several forms of glutathione were found as discriminating compounds in LE-treated plants. Glutathione is a strong antioxidant essential for antioxidant defence, xenobiotics detoxification, and maintenance of redox balance, among other processes [48].

In line with the non-enzymatic antioxidant system, several flavonoids have been strongly accumulated in the presence of LE. This extract has been reported to contain phenolic compounds, other antioxidant metabolites and signalling compounds [21]. Previous studies have revealed that the application of exogenous phenolics led to the elicitation of phenylpropanoids, which agrees with our results, as we found a substantial accumulation of these secondary metabolites, particularly flavonoids, considered effective ROS scavengers [49]. In fact, Zhang et al. [49] found a correlation between the application of exogenous phenolics (hesperidin and phenolic acids) and improved photosynthetic performance under stress conditions. 

Although the main effect of LE on maize was on the plant defence system, plant regulators were involved in response to the treatments. In fact, the phytohormones pattern was distinctly modified when LE was applied. Interestingly, several plant stress-related hormones accumulated in the LE-treated plants. In particular, brassinosteroids were positively regulated by LE. In this sense, the exogenous application of BRs seemed to mitigate the negative effect of Cu. Some authors suggested the involvement of BR in stimulating the antioxidant system and SOD, CAT and peroxidase (POX) activity in *Brassica juncea* plants subjected to Cu stress. Furthermore, these authors observed that BRs application also improved plant growth and photosynthesis of Cu-stressed plants [50]. 

Similarly, ABA derivatives, plant hormones that play an important role in plant responses to adverse conditions and that can interact with Cu as a micronutrient regarding its uptake and distribution, were found among the discriminant compounds [51]. Finally, the presence of signalling molecules in LE [21] could explain the elicitation of some pathways and the activation of some signalling cascade as ABA, which in turn is related to stoma activity, in agreement with our results. In this sense, the biosynthesis of benzoates, involved in the salicylic acid (SA) biosynthetic pathway, was stimulated when LE was applied. In fact, SA derivatives have been found in LE [21]. SA is a phytohormone used as a priming agent as it is implicated in several cellular processes during abiotic stresses [52], suggesting the involvement of the SA signalling cascade in mitigating the negative effect of Cu.

## 4. Materials and Methods

### 4.1. Duckweed Growth Conditions and Extract Preparation

Duckweed (*Lemna minor* L.) was grown in polyethylene trays (35 × 28 × 14 cm) according to a published procedure [2]. Trays were maintained at 23 ± 2 °C and light intensity of 100 μmol m^−2^ s^−1^ (light/dark photoperiod: 12/12 h). The culture media were renewed every two weeks with a nutrient solution consisting of 3.46 mmol L^−1^ KNO_3_, 1.25 mmol L^−1^ Ca(NO_3_)_2_·4H_2_0, 0.66 mmol L^−1^ KH_2_PO_4_, 0.071 mmol L^−1^ K_2_HPO_4_, 0.41 mmol L^−1^ MgSO_4_·7H_2_O, 0.28 mmol L^−1^ K_2_SO_4_, 1.94 μmol L^−1^ H_3_BO_3_, 0.63 μmol L^−1^ ZnSO·7H_2_O, 0.18 μmol L^−1^ Na_2_MoO_4_·2H_2_O, 1 μmol L^−1^ MnSO_4_·H_2_O, 21.80 μmol L^−1^ FeEDTA and 1 μmol L^−1^ CuSO_4_ [2]. 

About ten grams of duckweed were collected, copiously rinsed with water, and dried at 40 °C until constant weight. Then, 1 g of dried tissue was ground to powder using a mortar and pestle and extracted with 100 mL of deionized water (pH 7.0). The resulting suspension was left on a shaker overnight (100 rpm). After that, the suspension was filtered, and the liquid phase was brought to a final volume of 100 mL. This extract concentration-solution A-was designated as 1.00% LE (LE = lemna extract) and used directly to prime maize seeds or after suitable dilutions, as described in the following section.

### 4.2. Maize Growth Conditions and Seeds Priming

Maize seeds (*Zea mays* L., cv Belgrano) were sterilized with a sodium hypochlorite solution (0.25%) for 3 min and then washed repeatedly with deionized water. Next, the seeds were placed on Petri dishes (10 seeds per dish – diameter 14 cm – in triplicate for each treatment), filled with filter papers, and left in contact with 10 mL of solutions containing 0.00% LE (only water-Control), 0.05% LE (solution A diluted by a factor of 20), 0.50% LE (solution A diluted by a factor of 2), and 1.00% LE (solution A) of duckweed extract, respectively. The seeds were germinated in the dark at 23 °C for three days; after that, they were transferred into hydroponic solutions (six seedlings for each treatment in triplicate) containing a nutrient solution consisting of 2 mmol L^−1^ Ca(NO_3_)_2_·4H_2_O, 0.5 mmol L^−1^ MgSO_4_.7H_2_O, 0·7 mmol L^−1^ K_2_SO_4_, 0.1 mmol L^−1^ KCl, 0.1 mmol L^−1^ KH_2_PO_4_, 1 µmol L^−1^, H_3_BO_3_, 0.5 µmol L^−1^ MnSO_4_·H_2_O, 0.5 µmol L^−1^ CuSO_4_, 0.5 µmol L^−1^ ZnSO_4_·7H_2_O, 0.01 µmol L^−1^ (NH_4_)_6_Mo_7_O_24_ µ 4H_2_O, and 100 µmol L^−1^ Fe-EDTA. Seven days after growing under hydroponic conditions, the hydroponic solutions were added with 20 µM Cu SO_4_ except for the control group. In addition, some seedlings not primed with the LE extracts were treated with CuSO_4_ to ascertain the effect of copper in unprimed seeds. The seedlings were left to grow for a further week until they reached the second leaf stage, and plant material was collected and subjected to the following determinations.

### 4.3. Pn, gs, Evapotranspiration, Ci, and Plant Biomass 

After two weeks of growing under hydroponic conditions, leaf net photosynthesis (Pn), leaf transpiration rate (E), stomatal conductance (gs) and sub-stomatal CO_2_ concentration (Ci) were determined on four leaves for each treatment. Leaf gas exchange rates were measured using a portable IRGA (ADC-LCA-3, Analytical Development, Hoddesdon, UK) and a Parkinson-type assimilation chamber. Leaves were enclosed in the chamber and exposed to the same light as in the hydroponic system. Measurements were taken under steady-state conditions (after about 30 s); Pn, gs, and E were expressed on a leaf-area basis.

### 4.4. Determinations of Cu, H_2_O_2_ and MDA Contents

Two weeks after growing under hydroponic conditions, maize shoots (stem and leaves) were collected (three replicates for each treatment), washed and oven-dried at 80 °C till constant weight was reached. Then, 200 mg of leaves were digested with 8 mL of nitric acid (65% *v/v*) and 2 mL of hydrogen peroxide (30% *v/v*) at 90 °C for 90 min. Finally, the Cu concentration was quantified by Inductively Coupled Plasma (ICP) spectrometry [53].

As for MDA content determinations, 500 mg of the fresh shoot (three replicates for treatment) was extracted with 5 mL of 5% (*w*/*v*) trichloroacetic acid (TCA); then, the resulting suspension was centrifuged at 6000 rpm for 15 min. Next, 1 mL of the supernatant was added to 2.0 mL of 20% trichloroacetic acid (*w*/*v*) and 0.5% (*w*/*v*) thiobarbituric acid and heated at 95 °C for 20 min. After quick cooling, the MDA content was quantified spectrophotometrically [54].

The H_2_O_2_ content was determined in leaves by extracting the plant tissue (500 mg and three replicates for each treatment) with 5 mL of trichloroacetic acid 0.1% (*w/v*). The suspension was centrifuged at 6000 rpm for 10 min; then, 0.5 mL of the supernatant was used to estimate the H_2_O_2_ content spectrophotometrically at 390 nm [55].

### 4.5. Activity of the Antioxidant Enzymes SOD, APX and CAT

To assay superoxide dismutase (SOD) and catalase (CAT), maize leaves (1.0 g FW) were ground in liquid nitrogen and extracted with 50 mM KH_2_PO_4_/K_2_HPO_4_ (pH 7.8) (1:5 *w/v*). As for the ascorbate peroxidase (APX) enzyme, the same amount of maize leaves was extracted with 5 mL of a solution containing 0.1 M Tris-HCl, 2 mM ethylenediaminetetraacetic acid (EDTA), 1 mM dithiothreitol (DTT), and 1.5% polyvinylpolypyrrolidone (PVPP). The enzyme extracts were filtered through Miracloth and centrifuged at 6000 rpm for 20 min (4 °C). The total protein content in the extracts was determined according to Bradford [56].

Regarding the SOD assay, 2.60 mL of 50 mM KH_2_PO_4_/K_2_HPO_4_ (pH 7.8), containing 0.1 mM EDTA and 13 mM L-methionine, was added with 50 μL of the enzymatic extract, 300 μL of 75 μM nitro blue tetrazolium (NBT) and 30 μL of 2 μM riboflavin. The SOD activity was determined according to Beyer and Fridovich [57]. As for APX activity, 100 μL of enzyme extract, obtained as described above, were added to 1.7 mL of a solution containing 50 mM KH_2_PO_4_/K_2_HPO_4_ (pH 7.0), 100 μL of 0.50 mM ascorbic acid, and 100 μL of H_2_O_2_ (0.10 mM). The APX activity was determined according to Nakano and Asada [58]. Finally, for CAT activity, 100 μL of enzyme extract was added to 2 mL of 50 mM KH_2_PO_4_/K_2_HPO_4_ (pH 7.8) containing 10 mM H_2_O_2_. CAT activity was determined spectrophotometrically according to Aebi [59].

### 4.6. Untargeted Metabolomics

For each condition, three replicates were prepared. The samples were firstly extracted in an 80:20 methanol: water solution acidified with 0.1% (*v/v*) formic acid by using a homogenizer. The extracts were then centrifuged, and the supernatant filtered by 0.22 µm filters directly into vials for analysis. 

The untargeted metabolomics was carried out by ultra-high-pressure liquid chromatography coupled to a quadrupole-time-of-flight UHPLC-QTOF mass spectrometer from Agilent (Santa Clara, CA, USA), as previously reported [60]. Briefly, the chromatographic separation used a water-acetonitrile gradient elution (6% to 94% acetonitrile in 34 min) and an Agilent PFP column (50 mm × 2.1 mm, 1.8 μm). The mass spectrometric detection was carried out by SCAN acquisition (40,000 FWHM resolution) in positive polarity (range 100–1200 *m/z*).

The raw spectral data were processed according to a targeted ‘find-by-formula’ algorithm by the Agilent Profinder B.07 software (Agilent Technologies), following mass and retention time alignment. The comprehensive database Plant Metabolic Network PlantCyc 12.6 was used for compounds annotation [61] by combining monoisotopic mass, isotopes ratio and isotopes spacing, reaching a Level 2 of annotation (i.e., putatively annotated compounds) as reported by COSMOS Metabolomics Standards Initiative. Compounds [62] annotated in at least 75% of replicates within at least one treatment were retained for the analysis.

### 4.7. Statistics

For all the determinations on maize samples, the experiments were organized according to a completely randomized block design, with five treatments in triplicate. Statistical analysis was performed by analysis of variance (one-way ANOVA). According to Duncan’s test, significant differences were determined at *p* ≤ 0.05.

For metabolomics, through Agilent Technologies Mass Profiler Professional 12.6, the compound abundance was log2 transformed, normalized at the 75th percentile, and baselined against the median. Unsupervised hierarchical cluster analysis (HCA), based on fold-change values (Ward’s linkage and Euclidean distances), was used to naively describe patterns across treatments. Afterwards, a supervised class prediction model based on orthogonal projection to latent structures discriminant analysis (OPLS-DA) followed by Variable Importance in Projection (VIP) analysis was carried out by using SIMCA software (Umetrics, Malmo, Sweden). The VIP markers were considered significant when possessing a VIP score > 1.2. Then, these significative compounds were subjected to a fold-change analysis compared to the control and were exported to PlantCyc Pathway Tool (https://www.plantcyc.org, accessed on 08 March 2022) for biological interpretations [63,64].

## 5. Conclusions

In conclusion, this research revealed the ability of a plant extract obtained from duckweed, a wild aquatic species, to counteract the deleterious effects in maize plants caused by treatment with excessive amounts of copper, as indicated by the numerous physiological and growth parameters analysed. The protective action promoted by the duckweed extract can be related to its effectiveness in inducing antioxidant enzymes and its ability to increase the content of antioxidant, protective and hormonal molecules. In particular, the results showed that the most effective dosage for this purpose was 1.00% LE, although positive effects were also observed at the other doses studied.

In light of the above, this study shows that the adverse effects on crops due to copper can be counteracted by using biostimulants obtained from new plant extracts, thus indicating duckweed as a promising source of bioactive compounds. Finally, our results pave the way for sustainable management of abiotic stresses, particularly those related to copper pollution, through a viable approach that should not impact the environment. However, future studies in this direction are advised to complement the benefits highlighted in this work.

## Figures and Tables

**Table 1:** Shoot and root length and fresh weight (FW) recorded in control maize, samples treated with Cu alone and in combination with 0.05%, 0.50% and 1.00% LE.

	Shoot Length (cm)	Roots Length (cm)	Shoot FW (g plant^−1^)	Roots FW (g plant^−1^)
Control	23.2 (0.8) a	20.7 (1.7) ab	0.98 (0.24) b	0.200 (0.022) b
Cu	14.5 (0.9) b	8.3 (1.6) c	0.58 (0.09) c	0.129 (0.020) c
Cu + 0.05% LE	22.7 (1.4) a	18.8 (2.5) b	1.21 (0.26) ab	0.256 (0.045) ab
Cu + 0.50% LE	23.9 (1.8) a	23.2 (3.0) a	1.35 (0.15) a	0.289 (0.049) a
Cu +1.00% LE	22.6 (1.52) a	18.7 (1.0) b	0.83 (0.10) bc	0.264 (0.027) ab

Means followed by different letters were significantly different (*p* < 0.05), as indicated by one-way ANOVA followed by Duncan’s new multiple range test. The standard deviation (SD) is reported in brackets.

**Table 2:** Copper (Cu), hydrogen peroxide (H_2_O_2_), and malondialdehyde (MDA) found in control maize, samples treated with Cu alone and in combination with 0.05%, 0.50% and 1.00% LE.

	Cu (µg g^−1^ DW)	H_2_O_2_ (nmol g^−1^ FW)	MDA (nmol g^−1^ FW)
Control	18.4 (1.2) b	116.1 (5.0) d	21.6 (2.8) c
Cu	38.8 (7.1) a	211.6 (15.0) a	41.6 (4.3) a
Cu + 0.05% LE	35.1 (3.7) a	156.3 (19.4) bc	19.6 (1.5) c
Cu + 0.50% LE	36.3 (6.0) a	135.4 (20.4) cd	21.1 (3.7) c
Cu +1.00% LE	32.2 (4.5) a	179.6 (11.8) b	30.6 (3.1) b

Means followed by different letters were significantly different (*p* < 0.05), as indicated by one-way ANOVA followed by Duncan’s new multiple range test. The standard deviation (SD) is reported in brackets.

## Data Availability

Not applicable.

## Supplementary Materials

The following supporting information can be downloaded at: https://www.mdpi.com/article/10.3390/plants11192613/s1, Table S1: Metabolomics raw dataset of maize plants treated with 0.05% LE and 1.00% LE. The complete list of annotated metabolites, their composite mass spectra and individual raw abundance. Table S2: Discriminant metabolites identified by the Variable Importance in Projection (VIP) analysis following OPLS-DA modelling of the metabolome in maize leaves treated with 0.05% LE. Compounds were selected as discriminant by possessing a VIP score > 1.20, exported to the plantcyc omic viewer, and used for Figure 4. Table S3: Discriminant metabolites identified by the Variable Importance in Projection (VIP) analysis following OPLS-DA modelling of the metabolome in maize leaves treated with 1.00% LE. Compounds were selected as discriminant by possessing a VIP score > 1.20, exported to the plantcyc omic viewer and used for Figure 4.
